# A mechanism linking sleep with CSF‐to‐Blood toxins clearance

**DOI:** 10.1002/alz.085021

**Published:** 2025-01-03

**Authors:** Noam Alperin, Ritambhar Burman

**Affiliations:** ^1^ Department of Biomedical Engineering, University of Miami, Miami, FL USA; ^2^ Department of Radiology, miami, FL USA

## Abstract

**Background:**

Clearance of brain toxins occurs during sleep, although the mechanism remains unknown. Previous studies implied that the intracranial aqueductal cerebrospinal fluid (CSF) oscillations are involved, but no mechanism was suggested. The rationale for focusing on the aqueductal CSF oscillations is unclear. This study focuses on the cranio–spinal CSF oscillation and the factors that modulate this flow. We propose a mechanism where increased cranio–spinal CSF movements enhance CSF‐to‐blood metabolic waste clearance through the spinal CSF re‐absorption sites, by increased the transfer of toxins from the toxin‐ rich cranial CSF to the toxin‐poor spinal CSF. A recent study demonstrating that disturbed sleep impairs CSF‐to‐blood but not brain to‐CSF clearance, supports the fundamentals of our proposed mechanism.

**Method:**

Eight healthy subjects underwent phase‐contrast magnetic resonance imaging to quantify the effect of respiration on the cranio–spinal CSF oscillations. Maximal CSF volume displaced from the cranium to the spinal canal during each respiration and cardiac cycle were derived as measures of cranio–spinal CSF mixing level, Displaced CSF volume was measured during normal breathing (NB) and Slow deep abdominal breathing (SAB). Displaced CSF volume between the 3^rd^ and 4^th^ ventricl3es during normal breathing were also measured.

**Result:**

Transition from normal to slow and abdominal breathing resulted in a 56% increase in the maximal displaced CSF volume. Maximal change in the arterial–venous blood volume, which is the driving force of the CSF oscillations, was increased by 41% during slow abdominal breathings. Displaced CSF volume between the ventricles during each oscillation were about 10‐fold smaller than the discalced CSF volume between the ventricles.

**Conclusion:**

Cranio–spinal CSF oscillations are driven by the momentary difference between arterial inflow and venous outflow. Breathing modulates the CSF oscillation through changes in the venous outflow. The amount of toxins being transferred to the spinal canal during each respiratory cycle is significantly increased during slow and deeper abdominal breathing, which explains enhanced CSF‐to‐blood toxins clearance during slow‐wave sleep and poor clearance during disrupted sleep.

